# Activity-based epidemic propagation and contact network scaling in auto-dependent metropolitan areas

**DOI:** 10.1038/s41598-021-01522-w

**Published:** 2021-11-22

**Authors:** Nishant Kumar, Jimi Oke, Bat-hen Nahmias-Biran

**Affiliations:** 1 ETH Zurich, Future Resilient Systems, Singapore-ETH Centre, Singapore, 138602 Singapore; 2 Department of Civil and Environmental Engineering, University of Massachusetts, Amherst, MA 01003 USA; 3 Department of Civil Engineering, Ariel University, Ariel, 40700 Israel; 4 Department of Civil and Environmental Engineering, Massachusetts Institute of Technology, Cambridge, MA 02139 USA

**Keywords:** Diseases, Infectious diseases, Viral infection, Mathematics and computing, Applied mathematics

## Abstract

We build on recent work to develop a fully mechanistic, activity-based and highly spatio-temporally resolved epidemiological model which leverages person-trajectories obtained from an activity-based model calibrated for two full-scale prototype cities, consisting of representative synthetic populations and mobility networks for two contrasting auto-dependent city typologies. We simulate the propagation of the COVID-19 epidemic in both cities to analyze spreading patterns in urban networks across various activity types. Investigating the impact of the transit network, we find that its removal dampens disease propagation significantly, suggesting that transit restriction is more critical for mitigating post-peak disease spreading in transit dense cities. In the latter stages of disease spread, we find that the greatest share of infections occur at work locations. A statistical analysis of the resulting activity-based contact networks indicates that transit contacts are scale-free, work contacts are Weibull distributed, and shopping or leisure contacts are exponentially distributed. We validate our simulation results against existing case and mortality data across multiple cities in their respective typologies. Our framework demonstrates the potential for tracking epidemic propagation in urban networks, analyzing socio-demographic impacts and assessing activity- and mobility-specific implications of both non-pharmaceutical and pharmaceutical intervention strategies.

## Introduction

Efforts to contain the spread of the COVID-19 pandemic and mitigate its impacts on health and daily life have highlighted the need to better understand the propagation of epidemics across human networks. Cities, in particular, have been hit hard due to their population density and extensive mass transit systems^1^. Governments across the world responded to the pandemic by imposing non-pharmaceutical interventions, such as preventative measures (e.g. social distancing, handwashing, and face masks), lockdown policies (e.g. travel restrictions, school closures, and remote work), and testing (e.g. contact tracing and quarantine)^2–7^, and subsequently, pharmaceutical interventions (vaccine development and immunization)^8–13^. However, the results of these interventions have been mixed. Furthermore, immunization rates in most parts of the world are relatively low, due to financial and logistical challenges. To ensure the best outcomes, sophisticated tools are therefore required to enable decisionmakers to accurately predict the trajectory of the pandemic in their locales, optimize the distribution of limited vaccine supplies, and model the yet unknown effects of mitigating strategies^14^. Hence, agent-based models (ABMs) are critical to this effort^15–18^. Compartmental epidemiological models have also been harnessed to track the propagation of COVID-19^19–39^. Current modeling frameworks have been enhanced to account for super-spreading^37^ and population states, such as isolated^19,20,22,24–26,29,30,32,33^, hospitalized^19,20,25,26,29,33^, and asymptomatic infected^24,26,27,29,34^ cases as well as anti-vaccine behavior^40^.

Agent-based models capture the complexity of human mobility and social patterns more richly than the classical approaches^2,38,41,42^. Prior low-resolution agent-based models relied on broad approximations regarding the trajectories of the populations being represented^41^. With modern computational advances and the availability of mobile data, however, ABMs have demonstrated great potential in accurately tracking the spread of an epidemic at multiple levels, as they feature highly granular representations of agent movements^39,43^. Mesoscopic and microscopic transportation agent-based models, in particular, provide an even more detailed representation of the activity behavior within the physical network^44,45^. Only recently have these models been harnessed for the study of disease propagation^4,17,41,45,46^. Yet, there remains a research gap in frameworks that not only provide a detailed spatio-temporal representation of activity and mobility networks, but also allow for socio-demographic analyses of interventions. Furthermore, modeling efforts are specific to a given city or country, thus limiting the impacts of the insights they provide. Given the time and costs involved in developing these models, there is a clear need for urban simulation approaches that can produce valid results that are broadly applicable to cities sharing similar characteristics.

**Figure 1:**
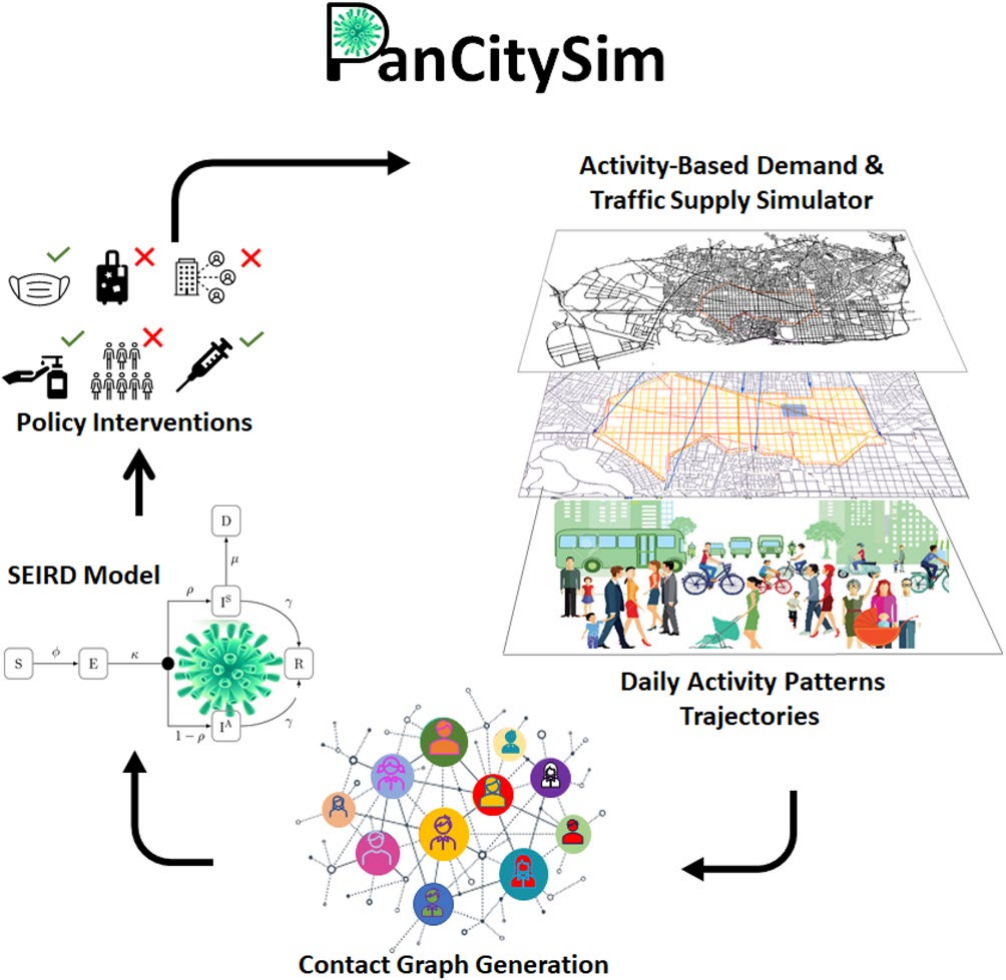
Overview of the PanCitySim framework.

To address these gaps, we propose a new framework, PanCitySim, which layers a fully stochastic and mechanistic agent-based epidemiological model onto highly time-resolved and spatially disaggregated daily person-trajectories Fig. 1. These trajectories are obtained from a microscopic travel demand and mesoscopic supply simulator, SimMobility^44,47,48^ that has been calibrated for two prototype cities, which represent sparse and dense automobile-dependent cities, respectively. We demonstrate that PanCitySim can be used to not only predict the spatio-temporal dynamics of an epidemic, but also potentially evaluate the impacts of a variety of interventions, such as employment-based or age-based restrictions, reduction in mass transit services also provide detailed contact-tracing and individual-level analyses of disease impacts. Importantly, we validate the outcomes using real-world observations from cities in each of the two typologies simulated.

## Methods

PanCitySim provides spatio-temporal COVID-19 propagation outcomes based on a high-resolution mobility simulation of person and vehicle movements in an average day for a full-scale city. We implemented PanCitySim for two prototype cities, which are simulated on their respective networks to provide multi-modal trajectories for each person at a five-minute resolution. From these activity-specific person-trajectories, we generated activity-based contact networks for the entire population and analyzed their scaling properties. We then simulated epidemic propagation via a Susceptible-Exposed-Infectious-Recovered-Deceased (SEIRD) epidemiological model on the contact network. The epidemiological model takes into account the latest measurements of relevant parameters, such as the incubation period, duration of infectiousness, and age-dependent likelihood of symptomaticity and mortality^4,45,49,50^. We validated our epidemic propagation predictions using actual infections and mortality observations from the relevant cities. Finally, we simulated a scenario where the transit network (including its corresponding contacts) is removed, and analyzed the outcomes on disease propagation. The authors confirm that all methods were carried out in accordance with relevant guidelines and regulations.

### Activity-based simulation model and city data

**Figure 2:**
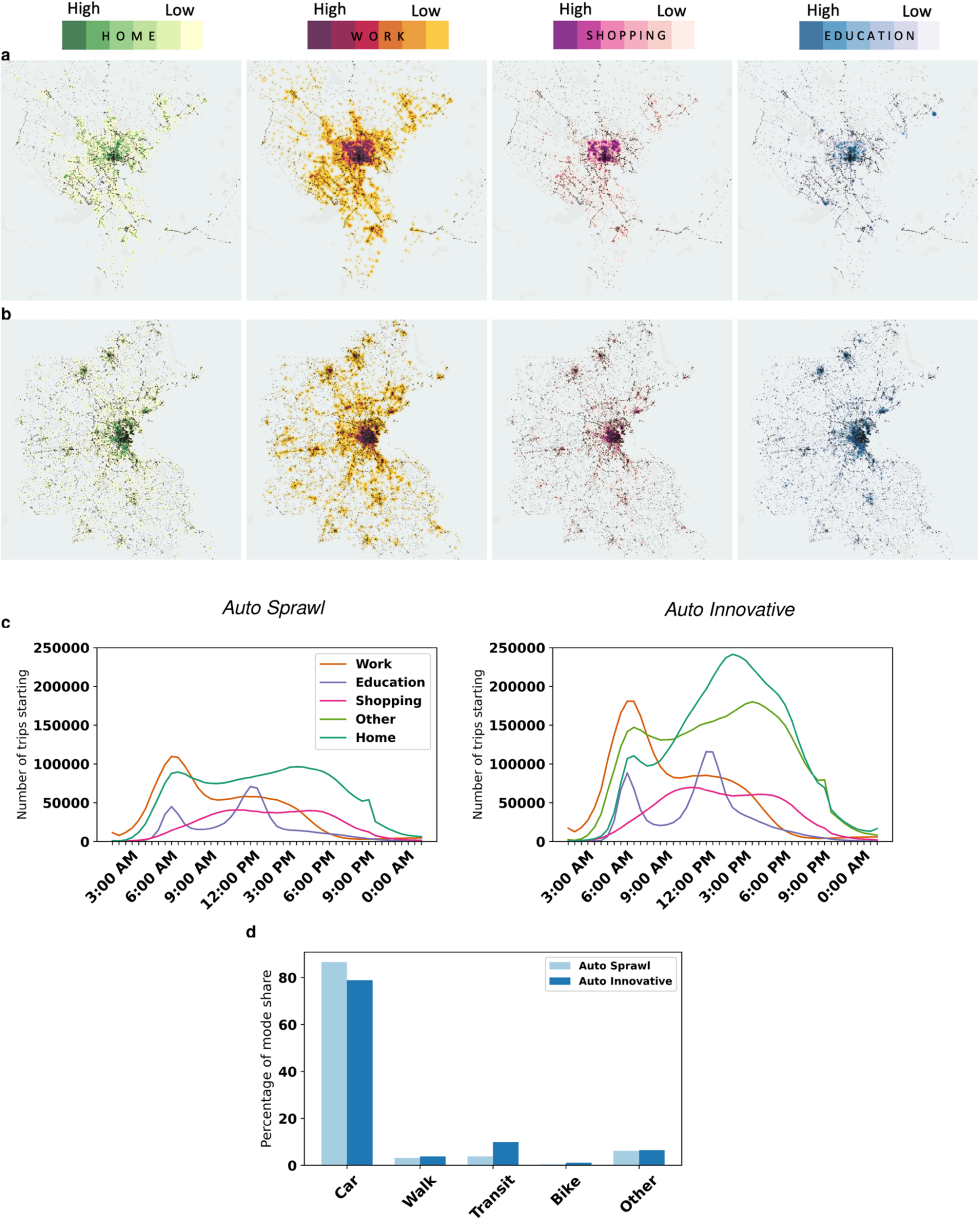
Activity and mobility patterns in two prototype cities. Spatial distribution of activity locations in (**a**) *Auto Sprawl*, and (**b**) *Auto Innovative*. The radii of heat maps are the same across both the cities; (**c**) Number of trips starting at various 30-minute intervals during the day; (**d**) Mode share for the two cities. Daily number of trips in *Auto Sprawl* is $9.72\times 10^6$, while in *Auto Innovative* it is $16\times 10^{6}$. Both cities have a similar trip generation rate of 3.5 per person.

A recent study identified 12 urban typologies based on 69 mobility, socio-demographic, environmental and network structure obtained from over 300 cities^51^. The two auto-dependent typologies consisting of cities largely in the US/Canada were used as the test beds for this study: *Auto Sprawl* (86% car mode share; e.g. Baltimore, Tampa, Raleigh) and *Auto Innovative* (78% car mode share; e.g. Washington D.C., Atlanta, Boston). *Auto Sprawl* typifies the lower-density US/Canada cities with low transit usage ($\sim$4%), while *Auto Innovative* consists of denser cities with an average of 11% transit mode share. *Auto Innovative* is also three times as populated on average, but only slightly denser, which indicates that *Auto Sprawl* cities have larger areas than those in *Auto Innovative*. The GDP per capita of *Auto Innovative* cities is 1.2 higher on average compared to that of *Auto Sprawl* cities. Given these key differences between the two typologies, prototype cities representing the population, land-use and mobility demand and supply outcomes in both typologies were synthesized^47^. Both prototype cities were built on actual road and transit networks, population microdata and land use categories from representative (or archetype) cities close to the centroid of their respective typologies. For *Auto Sprawl*, the archetype chosen was the Baltimore Metropolitan Area (population $2.77\times 10^{6}$, density $4.11\times 10^{2}$km$^{-2}$), while for *Auto Innovative*, it was Greater Boston (population $4.6\times 10^{6}$, density $5.09\times 10^{2}$km$^{-2}$). However, the demand and supply models for both prototype cities were calibrated to fit average typology values, in order to ensure representativeness of overall mobility outcomes. The spatio-temporal activity and mobility patterns of each city are shown in Fig. 2.

The simulation of the prototype cities was performed in SimMobility, an open-source platform for microscopic demand and mesoscopic supply dynamic traffic assignment modeling^44^. The cities were calibrated for modeshares, activity patterns and network speeds^47^. The inputs to the simulator are: land use, demographic and economic factors, as well as road and transit networks. A discrete choice modeling framework then simulates daily activity schedules (DAS) for each individual in a given synthetic population. Parameters for population (including age, gender, employment, vehicle ownership and household characteristics), land use and road/transit networks are obtained from a real-world archetype city in the typology. The DAS is a high level plan, including only important choices, which are translated into trip chains. Lower level choices are made during the day when those plans are executed. During the day, agents are either performing an activity (*Home*, *Work*, *Education*, *Shopping* or *Other*) or executing a trip. During an activity, agents are stationary at one location and then, at the beginning of each trip, agents further detail their plan. Once the detailed plan is made and the start time is reached, the supply simulator moves the agents accordingly. Given the path of each traveler, the supply simulator produces the actual movement trajectory of each heterogeneous vehicle type and pedestrian (passenger) movements, which are performed on the network to provide event-driven trajectories for each person. These trajectories are the final outputs of the simulator (SimMobility). The outputs of the traffic simulator serve as the inputs to the SEIRD model. As long as the spatio-temporal location of the individuals are produced as output, any traffic simulator can be used to produce the inputs for the SEIRD model. From the 5-minute person-trajectories, activity-specific contact graphs are constructed and transmission events are simulated.

### SEIRD model

We define the following states in our susceptible-exposed-infectious-recovered-deceased (SEIRD) model: susceptible ($\varvec{S}$), exposed, i.e. infected but not contagious ($\varvec{E}$), infectious and symptomatic ($\varvec{I}^{S}$), infectious but asymptomatic ($\varvec{I}^{A}$), recovered ($\varvec{R}$) and deceased ($\varvec{D}$). We assume that symptomatic cases are automatically quarantined by the end of the day. The transitions to each of these states are governed by the following probabilities:

1 $$\begin{aligned} \phi _{n,t}&= 1 - e^{\left( -\Theta \sum _m q_{m,t}\cdot i_{nm,t}\cdot \tau _{nm,t}\right) }\qquad \end{aligned}$$

2 $$\begin{aligned} \kappa _{n,d}&= 1 - e^{-\frac{1}{d_L}}\qquad \end{aligned}$$

3 $$\begin{aligned} \gamma _{n,d}&= 1 - e^{-\frac{1}{d_I}} \qquad \end{aligned}$$

4 $$\begin{aligned} \mu _{n,d}&= 1 - e^{-\frac{1}{d_{D}}}\qquad \end{aligned}$$

$\phi$ is the probability of infection, where $\Theta$ is a parameter to be calibrated. Using the mechanistic framework^15,45^, *q* is a measure of viral shedding rate [m$^3$] and *i* the contact intensity [1/m$^3$]. $\tau$ is the duration of contact. The indices are *m* (susceptible population), *n* (infectious) and *t* (time step). $\kappa$ is the daily probability of transitioning from exposed $\varvec{E}$ to infectious $\varvec{I}^{\{A,S\}}$ and $d_L$ is the incubation period^4^. Furthermore, $d_L \sim \text {Lognormal} (\mu = 1.62,\sigma ^2=0.42)$^50^. The median incubation period is taken as 5 days^50^. $\rho$ is the probability an infectious person is symptomatic. Thus, the transition to state $\varvec{I}^S$ is governed by the probability $\rho \kappa$. Further, $\alpha$ is the proportion of infectious people who are symptomatic, $\gamma$ is the daily probability that a person recovers ($d_I$ is the duration of infectiousness, also lognormally distributed) and $\mu$ is the mortality rate. Evidence suggests that $\rho$ and $\mu$ are highly age-dependent^4^. Given that case fatality rates (CFR) have been shown to differ significantly by age group, we use existing adjusted posterior mode estimates of the CFR, based on measurements over 41 days^49^. We assume the CFR is obtained from the CDF of an underlying exponential distribution governing the duration from onset of COVID-19 to death. Thus:

5 $$\begin{aligned} P(T > 41)&= 1 - e^{-41/d_{D}} = CFR \end{aligned}$$

The value of $d_{D}$ computed from the above equation is taken as the median of the lognormal random variate. Thus, the mean of the associated normal distribution is obtained by $\mu _{\ln d_{D}} = \ln (\hat{d}_{D})$. We also compute the variance of $d_{D}$ based on credible interval estimates^49^ $d_{D}$. Finally, we estimate the variance of the associated normal distribution, that is $\sigma ^{2}_{\ln d_{D}}$ by solving the transcendental equations relating the parameters of the lognormal distribution to those of the associated normal distribution. These parameters are summarized in Table 1.

**Table 1:** Age-dependent case fatality rate (CFR) parameters.

Age	Sample size	CFR (95% CI)	${{\hat{\mu }}_{\ln d_D}}$ (95% CI)	${{\hat{\sigma }}_{d_{D}}}$	${{\hat{\sigma }}_{\ln d_{D}}}$
0–9	4.160$\,\cdot 10^{2}$	2.600$\,\cdot 10^{-5}$ (3.120$\,\cdot 10^{-6}$, 3.820$\,\cdot 10^{-4}$)	6.782$\,\cdot 10^{7}$ (1.314$\,\cdot 10^{7}$, 1.073$\,\cdot 10^{5}$)	1.427$\,\cdot 10^{1}$	1.942
10–19	5.490$\,\cdot 10^{2}$	1.400$\,\cdot 10^{-4}$ (2.880$\,\cdot 10^{-5}$, 7.590$\,\cdot 10^{-4}$)	8.186$\,\cdot 10^{6}$ (1.424$\,\cdot 10^{6}$, 5.400$\,\cdot 10^{4}$)	1.259$\,\cdot 10^{1}$	1.830
20–29	3.619$\,\cdot 10^{3}$	6.000$\,\cdot 10^{-4}$ (3.170$\,\cdot 10^{-4}$, 1.320$\,\cdot 10^{-3}$)	1.508$\,\cdot 10^{6}$ (1.293$\,\cdot 10^{5}$, 3.104$\,\cdot 10^{4}$)	1.113$\,\cdot 10^{1}$	1.765
30–39	7.600$\,\cdot 10^{3}$	1.460$\,\cdot 10^{-3}$ (1.030$\,\cdot 10^{-3}$, 2.550$\,\cdot 10^{-3}$)	5.277$\,\cdot 10^{5}$ (3.979$\,\cdot 10^{4}$, 1.606$\,\cdot 10^{4}$)	1.024$\,\cdot 10^{1}$	1.720
40–49	8.571$\,\cdot 10^{3}$	2.950$\,\cdot 10^{-3}$ (2.210$\,\cdot 10^{-3}$, 4.220$\,\cdot 10^{-3}$)	2.087$\,\cdot 10^{5}$ (1.853$\,\cdot 10^{4}$, 9.695$\,\cdot 10^{3}$)	9.538	1.656
50–59	1.001$\,\cdot 10^{4}$	1.250$\,\cdot 10^{-2}$ (1.030$\,\cdot 10^{-2}$, 1.550$\,\cdot 10^{-2}$)	3.408$\,\cdot 10^{4}$ (3.960$\,\cdot 10^{3}$, 2.625$\,\cdot 10^{3}$)	8.089	1.548
60–69	8.583$\,\cdot 10^{3}$	3.990$\,\cdot 10^{-2}$ (3.410$\,\cdot 10^{-2}$, 4.550$\,\cdot 10^{-2}$)	7.121$\,\cdot 10^{3}$ (1.182$\,\cdot 10^{3}$, 8.804$\,\cdot 10^{2}$)	6.915	1.423
70–79	3.918$\,\cdot 10^{3}$	8.610$\,\cdot 10^{-2}$ (7.480$\,\cdot 10^{-2}$, 9.990$\,\cdot 10^{-2}$)	2.201$\,\cdot 10^{3}$ (5.274$\,\cdot 10^{2}$, 3.896$\,\cdot 10^{2}$)	6.121	1.296
$\ge 80$	1.408$\,\cdot 10^{3}$	1.340$\,\cdot 10^{-1}$ (1.120$\,\cdot 10^{-1}$, 1.590$\,\cdot 10^{-1}$)	1.038$\,\cdot 10^{3}$ (3.452$\,\cdot 10^{2}$, 2.368$\,\cdot 10^{2}$)	5.652	1.195

The number of days from onset to death $d_{D}$ is assumed to be lognormally distributed. The CFR (95% CI) and sample size for respective age categories are obtained from^49^. The reported CFR values are taken as the CDF of an exponential distribution with mean rate of occurrence $\frac{1}{d_{D}}$ at day 41. The computed $d_{D}$ is then taken as the median of the associated lognormal distribution, from which the parameters of the associated normal distribution, ${\hat{\mu }}_{\ln d_{D}}$ and ${\hat{\sigma }}_{\ln d_{D}}$, are computed.

#### Contact intensity

The transmission probability is explicitly dependent on the separation distance and shedding rate^15^. With regard to distance, the contact intensity *i* has an inverse cube relationship. For simplicity, we assume the shedding rate ($q_{0}$) remains constant, but estimate the separating distance $d_{n,m}$ at time step *t* between two persons *n* and *m* at a given physical node *V* based on locations which are assumed to be normally distributed about the respective node center. We then calibrate for a fixed contact intensity $i_{0}$, which we then scale by $\frac{1}{d_{nm}^{3}}$ per node and time step. For agents in transit, we partition the vehicles into squares, randomly assign passengers to each square partition, and estimate the expected distance between randomly selected passenger pairs. A detailed description of the distance computation between individuals who share space in transit and those who share space while performing an activity are presented in Supplementary Sections 2.1 and 2.2, respectively. When agents are at home, we use the expected distance between inhabitants based on the average square footage of homes in the city. Thus, the probability of transmission is given by:

6 $$\begin{aligned} \phi _{n,t} = 1 - \exp \left\{ -\Theta q_0 i_{0} \sum _m \frac{1}{d_{n,m}^{3}} \cdot \tau _{nm,t} \right\} \end{aligned}$$

#### Contact network generation

A contact network is a collection of several contact graphs at different times of day. In this framework, we generate contact graphs at 5-minute time steps for the entire population using activity-based simulated trajectories from calibrated models of the two cities. The contact graph, at any 5-minute time window, is created by a union of three subgraphs—*Home*, *Activity* or *Transit*. The *Home* graph consists of agents who share a home during the time window. The *Activity* graph comprises individuals who are performing an activity (*Work*, *Education*, *Shopping* or *Other*). The *Transit* graph consists of individuals who are traveling in a bus or train or waiting for the same. We assume that motorists make no contacts while en route to their destinations, hence they are not modeled in our contact graph. To facilitate an efficient representation of the contact graphs, we employ a hub-and-spoke transformation, which leverages on the sparseness of the graphs (Supplementary Figure 7).

#### Calibration

To find $\Theta$, we calibrate the SEIRD model to achieve a basic reproductive number (average number of secondary cases caused by an infected person in the early stage of the epidemic) of $R_0 = 2.5$ using:

7 $$\begin{aligned} R_0 = \frac{1}{X}\sum _m^X\sum _n^S \left( 1 - e^{-\Theta '\sum _m \tau _{nm}}\right) \end{aligned}$$

where $\Theta ' = \Theta {q}_{0}{i}_{0}$, and *X* is the set of index cases while *S* the set of secondary cases^15^. We define $R_0$ as the average number of new infections per initially infected person. We calibrate $R_0$ over a period of 5 days on the full population, as this is the median duration of incubation^50^. Post calibration, $R_0$ was equal to $2.43 \pm 0.14$ in case of *Auto Sprawl* and $3.19 \pm 0.16$ in case of *Auto Innovative* using 30 samples for both cases (errors are 95% confidence intervals). The five-day average of $R_{t}$ (daily reproductive rate), however was 0.77 in both cities. While calibrating, we found that a universal value of $\Theta '$ is not possible. For a given value of $\Theta '$, the variance in the value of $R_0$ decreases with an increase in $I_0$. This behavior shown in Fig. 7c. In order to arrive at a stable value calibrated $\Theta '$, the $I_0$ was set to 1000 for calibration.

## Results

### Analysis of contact network structure and scaling properties

**Figure 3:**
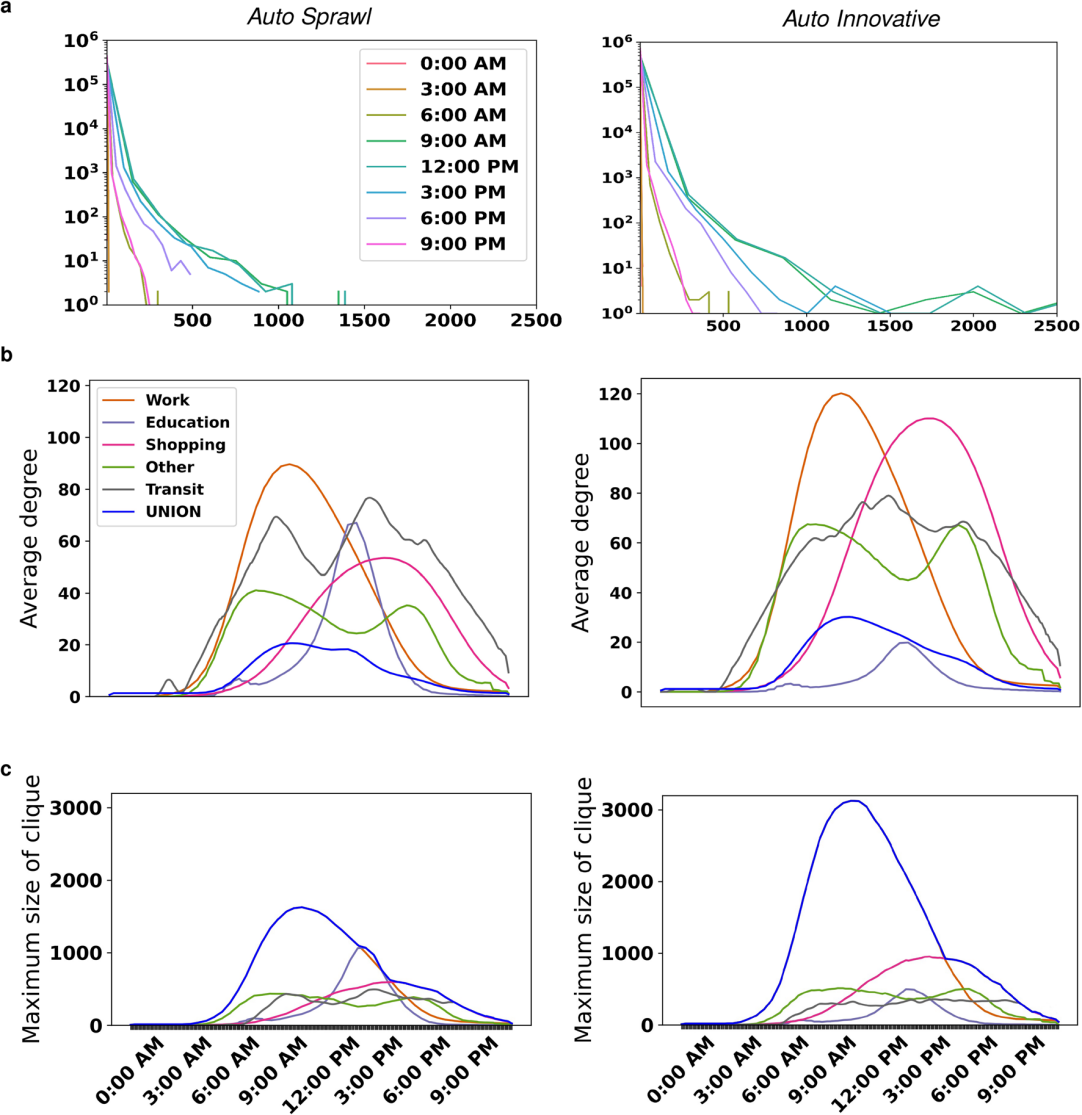
Contact network structure. The *Union* graph combines the contact networks from all activity types in a given day. (**a**) Degree distributions of the contact network (*Union* graph) for selected times of day; (**b**) The average degree $\langle k\rangle$ for both cities are shown over the course of the day; (**c**) Diameter (maximum clique size). During work hours, the maximum clique size of the *Union* graph is due to the *Work* graph; post work hours, this is due to the *Shopping* graph. Plots are smoothed using a moving average of six time steps.

We generated an activity-based contact network for the population in each city every 5 minutes (see Methods). The *Union* contact network comprises all activities for each individual: *Work*, *Education*, *Shopping*, *Other* and *Transit*. The *Transit* activity comprises waiting and the duration of time spent on a bus or train. The spatial resolution of each contact is at the node level (activity location) or at transit vehicle (bus/train) partitions. Figure 3a shows the degree distribution for the *Union* contact network at selected times in both cities. Further, we plot the average degree $\langle k \rangle$ for each activity contact network, including the *Union* (Fig. 3b). We observe that *Work* is responsible for the maximum average number of contacts, peaking at 10 AM with 90 contacts in *Auto Sprawl* and 120 in *Auto Innovative*. In *Auto Sprawl*, the activity responsible for the second highest average number of contacts per time step was *Transit* with 70 contacts on average at morning peak (8 AM) and almost 80 contacts on average at evening peak (3 PM). In *Auto Innovative*, however, the activity responsible for second greatest peak contact average was *Shopping* with 110 contacts per person between 4 and 5 PM. The maximum number of people who are in contact at any given time of the day is represented by the maximum clique size of the contact graph, as shown in Fig. 3c. On average, the maximum number of contacts created in *Auto Sprawl* is more than 1600 between 10 and 11 AM, while for *Auto Innovative* the maximum number of contacts is twice as high during the same time.

In order to gain further insights into the contact network structure, we analyzed the complementary cumulative distribution functions (CCDF) for each 5-minute time step by activity (Supplementary Figure 8). There appear to be no identifiable patterns in the *Union* contact graphs, but we hypothesized that the *Work* contact network follows a Weibull distribution $p_{k } \propto e^{\left( -\lambda k \right) ^{\beta }}$, while the *Transit* contact network obeys the power law, $p_{k} \propto k^{-\alpha }$. Contact networks for *Shopping* and *Other* activities appear to follow an exponential distribution $p_{k} \propto e^{-\lambda k}$. In order to test these null hypotheses, we conducted Kolmogorov-Smirnov (KS) tests to determine if alternative distributions provide better fits^52^. The KS tests produce p-values based on loglikehood ratios between the alternative and null fits. In all cases, there was no sufficient evidence to reject the null hypothesis. (Fitted CCDFs are shown in Supplementary Figs. 9a and 9b).

The fitted Weibull parameters for *Work* diverge between both cities. The scale-free *Transit* contact graphs were fitted to $p_{k} \propto k^{-{\hat{\alpha }}}$, with a cut-off at $k=150$. Between 8 AM and 8 PM, ${\hat{\alpha }} \approx 1.4$ in both cities. For *Shopping*, which follows an exponential distribution, ${\hat{\lambda }}$ is similar for both cities at the same time periods. In the case of *Other*, however, while the trends in *Auto Sprawl* and *Auto Innovative* are similar, ${\hat{\lambda }}$ tends to be higher in *Auto Sprawl*. A summary of all the fitted parameters for the distributions governing *Work*, *Shopping*, *Other* and *Transit* is given in Table 2 (corresponding plots are shown in Supplementary Figure 9c).

The fitted distributions are assumed to be valid from a minimum degree $\hat{k}_{\min }$, which is determined by minimizing the KS-distance between observed and predicted values based on the power law fit. While this $\hat{k}_{\min }$ is biased, it facilitates a consistent comparison between candidate distributions^53^. Other alternative distributions considered were the lognormal and truncated power law. Notably, for both *Transit* contact networks, the parameter $\hat{k}_{\min }$ is largely time-invariant, averaging 2.4 in *Auto Sprawl* and 2.2 in *Auto Innovative* (Supplementary Figure 9d). This indicates that the power law takes effect with a minimum of two or three contacts in each city’s transit network.

**Table 2:** Estimated parameters for fitted degree distributions of activity-based contact networks in *Auto Sprawl* (AS) and *Auto Innovative* (AI).

Activity	Work						Shopping				Other			
Distribution	Weibull: $p_{k } \propto e^{\left( -\lambda k \right) ^{\beta }}$						Exponential: $p_{k} \propto e^{-\lambda k}$				Exponential: $p_{k} \propto e^{-\lambda k}$			
Parameters	${\hat{\lambda }} (\times 10^{1} )$		${\hat{\beta }} (\times 10^{-1} )$		${\hat{k}}_{\min }$		${\hat{\lambda }} (\times 10^{-2} )$		${\hat{k}}_{\min }(\times 10^{2} )$		${\hat{\lambda }} (\times 10^{-2} )$		${\hat{k}}_{\min } (\times 10^{1} )$	
	AS	AI	AS	AI	AS	AI	AS	AI	AS	AI	AS	AI	AS	AI
08:00 AM	26.4	308	2.2	1.8	121	84	10.6	7.0	0.1	0.6	1.9	1.5	4.5	16
09:00 AM	68.8	701	2.0	1.7	158	109	6.5	4.5	0.2	0.5	1.4	1.4	8.9	18
10:00 AM	9.1	581	2.3	1.7	173	117	3.9	2.9	0.3	0.8	2.0	1.4	4.2	16
11:00 AM	13.1	540	2.2	1.7	169	119	2.5	2.0	0.6	1.2	1.9	1.4	5.2	17
12:00 PM	39.5	279	2.1	1.8	174	119	1.9	1.5	0.9	1.7	2.1	1.6	4.5	14
01:00 PM	44.4	139	2.1	1.9	150	109	1.8	1.2	0.7	1.9	2.3	1.6	4.2	15
02:00 PM	5.5	58	2.4	2.0	132	83	1.4	1.1	1.3	2.1	2.2	1.8	4.6	14
03:00 PM	27.2	95	2.2	2.0	105	120	1.4	1.1	1.3	2.1	1.9	1.7	6.6	14
04:00 PM	0.9	255	3.0	1.9	74	112	1.3	1.1	1.4	2.1	2.2	1.5	4.3	16
05:00 PM	0.1	411	4.2	1.9	41	77	1.3	1.1	1.5	1.8	2.1	1.3	4.3	19
06:00 PM	0.1	570	4.4	2.0	45	40	1.4	1.2	1.5	1.9	1.9	1.3	4.7	15
07:00 PM	0.1	567	5.3	2.1	15	28	1.9	1.4	0.6	1.7	1.9	1.6	5.9	15
08:00 PM	0.7	9570	4.0	1.8	14	13	2.2	1.8	0.6	1.4	3.6	2.3	2.4	10
09:00 PM	0.4	784	4.5	2.1	9	10	2.9	2.6	0.7	0.9	4.6	3.6	2.1	8

**Activity**	*Transit*													
**Distribution**	Power law: $p_{k} \propto k^{-\alpha }$													
**Parameters**	${\hat{\alpha }}$		${\hat{\sigma }} (\times 10^{-2} )$		${\hat{k}}_{\min }$									
	AS	AI	AS	AI	AS	AI								
08:00 AM	1.4	1.6	2.6	4.0	2	4								
09:00 AM	1.4	1.5	2.8	3.4	2	3								
10:00 AM	1.5	1.4	3.4	2.3	3	2								
11:00 AM	1.4	1.3	2.4	2.2	2	2								
12:00 PM	1.4	1.4	2.4	2.6	2	2								
01:00 PM	1.4	1.4	2.7	2.7	2	2								
02:00 PM	1.5	1.4	3.5	2.7	3	2								
03:00 PM	1.4	1.4	3.1	2.6	3	2								
04:00 PM	1.4	1.4	2.6	2.6	2	2								
05:00 PM	1.5	1.4	3.2	2.1	3	2								
06:00 PM	1.5	1.4	3.5	2.4	3	2								
07:00 PM	1.4	1.3	2.5	2.0	2	2								
08:00 PM	1.4	1.3	2.8	2.1	2	2								
09:00 PM	1.0	1.2	5.5	1.3	10	2								

### Spatio-temporal evolution and activity-based impacts

**Figure 4:**
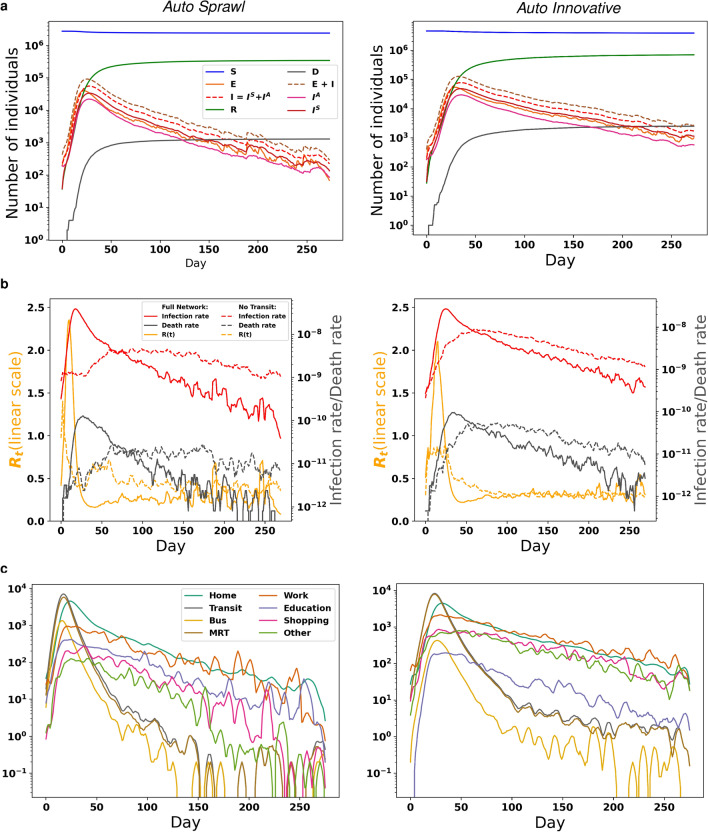
Simulated evolution of COVID-19 across two prototype cities. (**a**) Log y-axis of population states. (**b**) Time-dependent (daily) reproductive rate $R_{t}$, infection and mortality rates. (**c**) Daily transmissions by activity. The curves are smoothed with a moving average over 5 days. The infection rate and death rate are expressed per 100,000 persons.

We calibrated *Auto Sprawl* and *Auto Innovative* to fit the expected early dynamics of COVID-19^4,54–56^ (see Methods). We considered the basic reproductive rate $R_{0}$ (average number of secondary infections per index case), which ranged from 2.29 to 2.57 in *Auto Sprawl*, and from 3.03 to 3.35 in *Auto Innovative*. Given the uncertainty about $R_{0}$, we also considered the time-dependent reproduction number $R_{t}$ (average number of new daily infections per infectious individuals). We computed the five-day average of $R_{t}$ as a measure of the early propagation of the epidemic, which was 0.77 in both cities. Given these starting assumptions, we simulated the epidemic for 270 days in both cities (Fig. 4a). The peak number of infections occurred at day 27 in *Auto Sprawl* and at day 34 in *Auto Innovative*, dissipating after 150 days and 250 days, respectively. At the peak, there were $9.21\times 10^{4}$ infections (both exposed and infectious individuals) in *Auto Sprawl* and 1.38 times more infections in *Auto Innovative* (Fig. 4b).

We plot the daily infection and mortality rates, along with $R_{t}$ in Fig. 4c. The infection rate is given by the number of daily new infections as a proportion of the entire population. The mortality rate is defined as the number of daily deaths also with respect to the entire population. While both *Auto Sprawl* and *Auto Innovative* had similar early onset dynamics, we observe that disease propagation diverged rapidly between both cities. In *Auto Sprawl*, $R_{t}$ peaked at day 9, with an average rate of change of $1.78\times 10^{-1}$ day$^{-2}$ from onset to peak. Post-peak, however, the rate of change is $-9.61\times 10^{-3}$ day$^{-2}$. Furthermore, the slope of the infection rate from onset to peak (day 18) in *Auto Sprawl* is $5.15\times 10^{2}$ day$^{-2}$. Post-peak, the rate is $4.25\times 10^{1}$ day$^{-2}$. In *Auto Innovative*, however, $R_{t}$ peaked at day 13, with a slope steeper than that of *Auto Sprawl* by a factor of 1.1. $R_{t}$ then dissipated 2.4 times slower compared to *Auto Sprawl*. The infection rate peaked on day 26 in *Auto Innovative* (in twice the amount of time as in *Auto Sprawl*), and dissipated at a rate of $-5.44\times 10^{1}$ day$^{-2}$. From onset to peak, the mortality rate is 1.34 day$^{-2}$ in both cities, peaking on day 28 *Auto Sprawl* and on day 34 in *Auto Innovative*.

Observing epidemic propagation by activity (Fig. 4c), we find that at the onset, *Transit* activity was responsible for most of the transmissions, moreso trains than buses. At the peak of infection, *Transit* remained responsible for the greatest share of transmissions in *Auto Sprawl*, while *Home* and *Work* had the greatest share in *Auto Innovative*. In the latter stages, we find that the greatest share of infections occurred during *Work*. *Education* was also significant in *Auto Innovative*, while *Shopping* was responsible for a sizable number of infections in *Auto Sprawl*. The lowest impact activity was *Bus* transit. We note that the trajectory of the epidemic can also be analyzed across socio-demographic dimensions (such as age (Supplementary Figure 3)), which are available for the synthetic populations. Furthermore, as discussed in the section on Methods, the epidemiological model in PanCitySim has age-specific parameters. With this level of granularity, vulnerable demographics can be identified and relief operations and interventions targeted accordingly.

**Figure 5:**
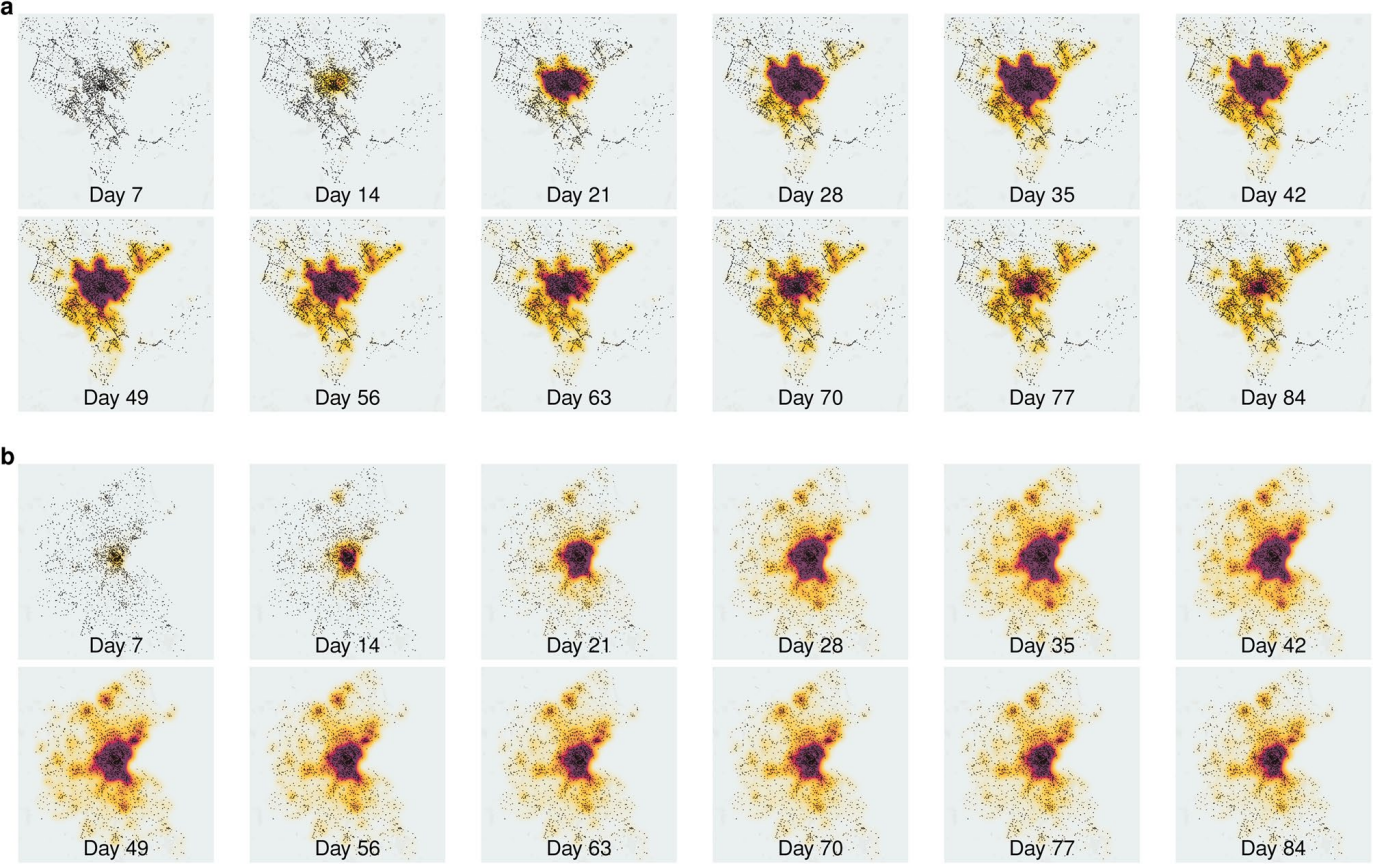
Spatial evolution of COVID-19. Heatmap of infected (exposed and infectious) individuals every seven days: (**a**) *Auto Sprawl*; (**b**) *Auto Innovative*. As expected, the prevalence is greatest in city centers, regardless of the location of index cases. In *Auto Innovative*, the hubs are evident.

We observe the spatial evolution of the epidemic in both cities (Fig. 5). In *Auto Sprawl*, at the onset of the epidemic, we identify several hubs, mostly located outside of the city center. These quickly migrate to the city center by the second week, becoming stronger there, with the peak of the disease being observed in the sixth week. In *Auto Innovative*, a denser city with greater transit usage, the onset of the epidemic begins in city center, grows rapidly and spreads out to the suburbs. The peak of epidemic is observed between weeks 5 and 7, after which there is a gradual decline of in the numbers throughout the city. We observe that towards the end of the simulation, the rate of decline in the number of cases is much slower for *Auto Innovative* compared to *Auto Sprawl*. At the end of our simulation period, *Auto Innovative* had around 10 times more active cases compared to *Auto Sprawl*.

### Impact of transit network on disease propagation

To understand the extent of the impact of the physical transit network on the propagation of the epidemic, we removed the *Transit* activity from the contact network construction in both *Auto Sprawl* and *Auto Innovative*. Under this scenario (*No Transit*), individuals could no longer travel via transit and thus had to choose an alternative mode or change their activity. In real-world circumstances, the majority of travelers in these types of cities would elect to travel by private car as single passengers, and thus not contribute to disease propagation while traveling. We then compared the SEIRD model outputs and time-dependent reproductive rates in the full-network and no-transit cases (Fig. 4b).

The results indicate that $R_{t}$ peaked on the first day in *Auto Sprawl* (the average value in the first five days was 1.40), dissipating at the rate of $-7.65\times 10^{-3}$ day$^{-2}$. The removal of transit thus effectively dampened the reproduction of the epidemic from the onset in *Auto Sprawl*. In *Auto Innovative*, $R_{t}$ peaked at day 25 (compared to day 13 in the full case), but its slope was five times smaller than in the full network. Thus, the force of the epidemic was also lessened in *Auto Innovative* due to transit removal.

This effect is even more apparent when we consider the infection rates. In *Auto Sprawl*, the infection rate peaked at day 86 with 1.49 $\times 10^3$ infections/day—a 98% decrease from the peak rate (day 19) in the full-network case. The slope of the infection rate (onset to peak) similarly decreased by 98% when transit was removed. Meanwhile, in *Auto Innovative*, the infection rate peaked at day 61 with 4.54 $\times 10^3$ infections/day as a result of transit removal—a decrease of 64% from the full-network peak (day 18). In a similar trend, the rate of change decreased by 84% in the no-transit case, when compared to the full network.

The removal of transit also dampened the force of mortality, as daily deaths peaked on day 62 in *Auto Sprawl* (compared to day 28 in the full-network network) and on day 78 in *Auto Innovative* (compared to day 34 in the full-network case). These results highlight the importance of modeling the *Transit* contact network in detail, and the central role that public transportation played in spreading the virus, especially in the early stages of the disease outbreak.

### Sensitivity analyses and validation

**Figure 6:**
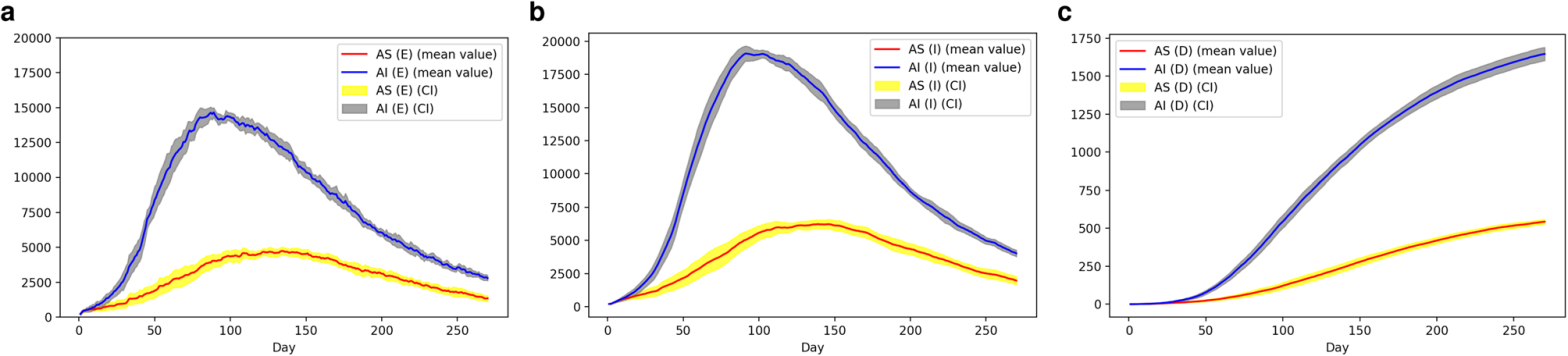
Stochasticity of PanCitySim Mean and 95% confidence intervals of predictions for Exposed cases (*E*), Infectious cases ($I_a+I_s$), and Deaths (*D*) respectively, based on 10 runs of PanCitySim for the full population for each prototype city: *Auto Sprawl* (AS) and *Auto Innovative* (AI).

As the dynamics of the COVID-19 are yet to be fully understood, with several possible assumptions including, but not limited to, re-infections^57^, multiple strains of the virus^58^ and multiple-outbreaks^59^, the simulation results presented here are just one realization out of numerous possible outcomes of the initial parameters. However, in generating our results, we ran the simulator ten times on the full population in each city. Figure 6 shows the mean and 95% confidence intervals for various outputs of the model.

**Figure 7:**
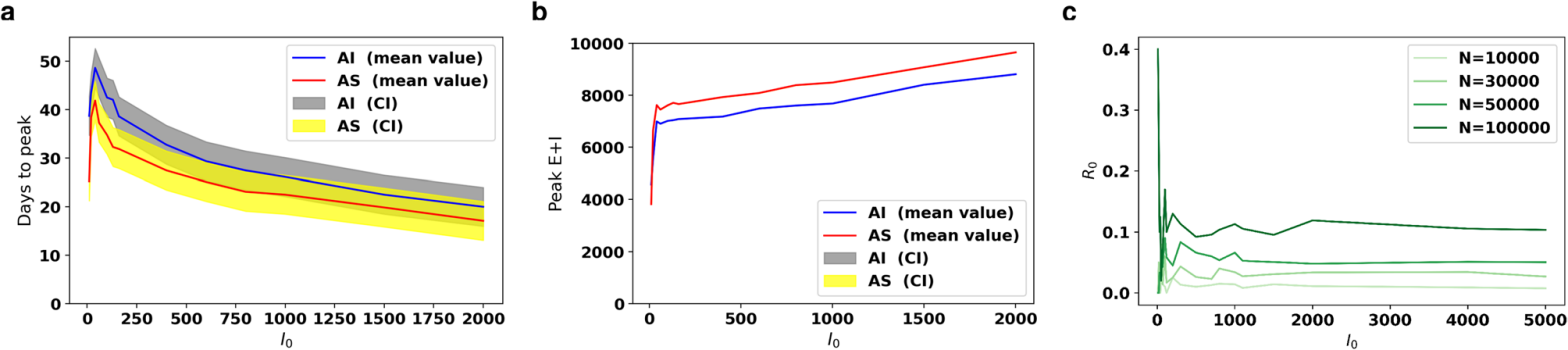
Sensitivity analysis of PanCitySim The number of initial infections ($I_0$) was varied and the simulation was run 50 times using a subset of population(100,000 households). The mean plots for (**a**) number of days to peak and (**b**) size of peak are shown vs $I_0$. We observe that higher number of initial infections results in bigger peaks which are achieved earlier. (**c**) shows a plot of of $R_0$ vs $I_0$ (for a fixed value of $\Theta '$). We observe that smaller values of $I_0$ result in a high stochasticity in the value of $R_0$. This observation is valid for various sample size of individuals(*N*).

Thus, we analyzed the sensitivity of three key variables (number of days to reach infection peak; peak number of infections and the basic reproductive rate) to changes in the initial number of infections ($I_{0}$) in both prototype cities. The results (Fig. 7) are based on a population sample comprising 100,000 households randomly selected from each simulated prototype city (*Auto Sprawl* and *Auto Innovative*). We varied the number of initial infections ($I_0$) and ran the simulation 50 times in each case. The sensitivity patterns of $I_0$ were similar in both prototype cities. We observe in Fig. 7a,b that a higher number of initial infections results in a larger and more rapid evolution of the epidemic.

**Figure 8:**
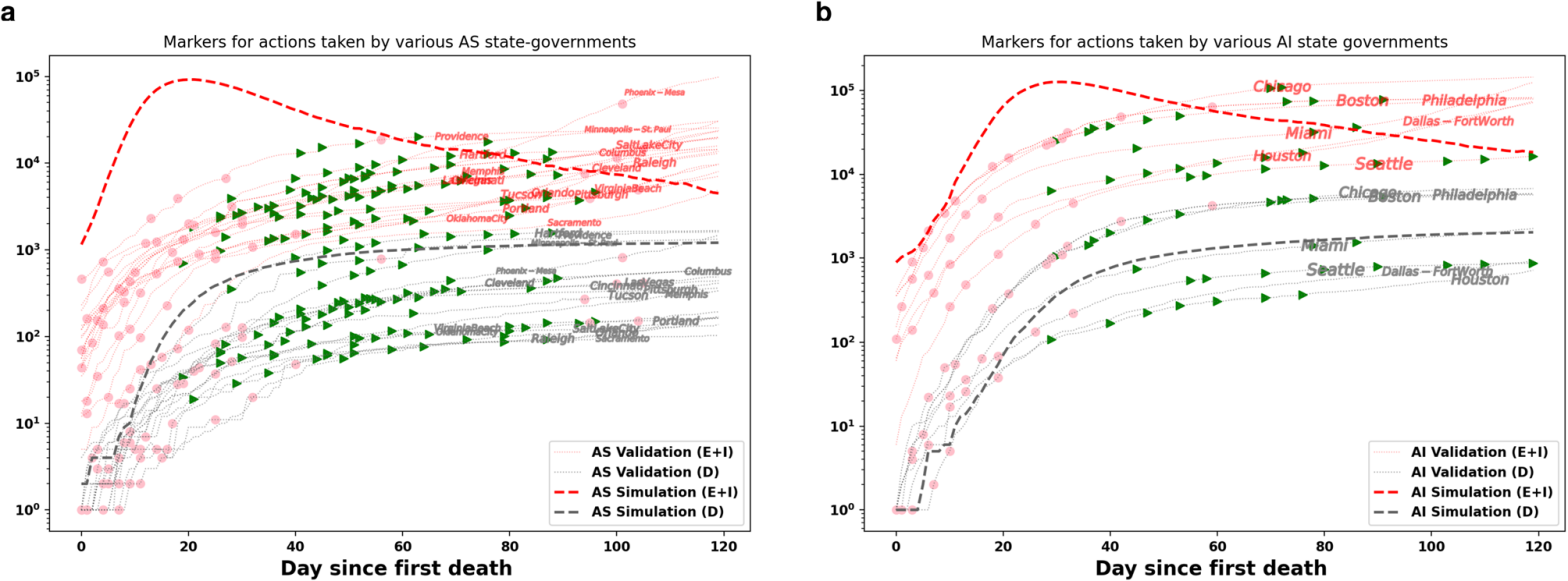
**Infections and deaths in archetype cities.** We compare simulation results to reported data on cases and mortalities. (**a**) *Auto Sprawl* (simulation) compared to other U.S. metropolitan areas in the same typology (e.g. Baltimore, Austin, Indianapolis). (**b**) *Auto Innovative* (simulation) compared to other U.S. metropolitan areas in the same typology (e.g. Boston, Chicago, San Francisco). Validation start dates were matched with earliest record of the first death. Data were obtained from the New York Times repository^60^ and the JHU CSSE COVID-19 Data repository^61^ summarizing the policy decisions. The restrictions put in place are represented by the red dots and the relaxation/lifting of the restrictions are represented by green triangles.

**Figure 9:**
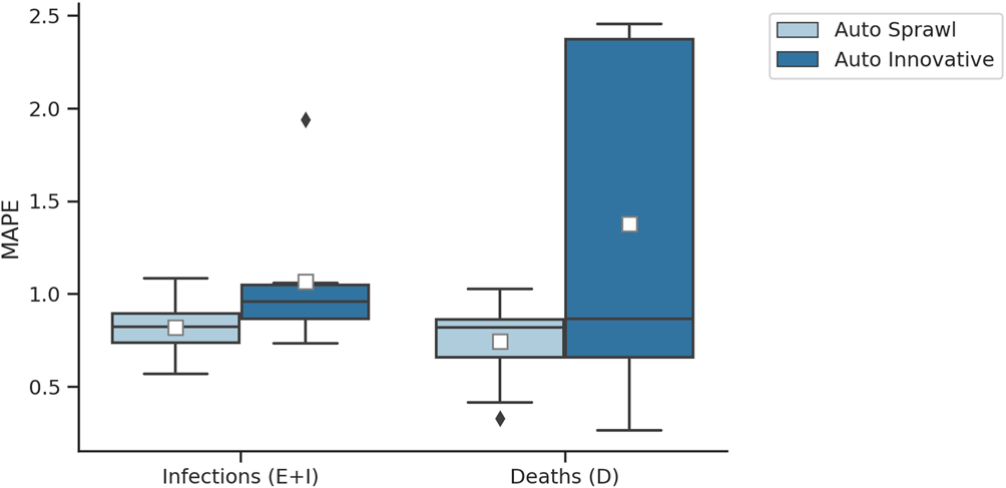
Boxplots showing the mean absolute percentage errors (MAPE) for the simulation output with respect to the observations from the cities in each typology. The MAPE is computed for exposed and infectious cases ($E+I$) and recorded deaths (*D*). The baseline is the day since the first death reported in each city.

We compare the simulation outputs (predicted infections and deaths) of PanCitySim to those of example cities from the respective typologies (Fig. 8). Given that our simulations are based on a no-intervention scenario, we also assess the dates of policy interventions put in place by respective governments in each city. First, we note that either a smaller or a delayed peak of the epidemic is seen in both cities compared to the respective simulated output. This can be attributed to the restrictions put in place at the start of the epidemic (represented by the red dots). Second, we observe an increase in the number of infections once those restrictions are relaxed or lifted (represented by green triangles). These two trends are more obvious in case of *Auto Sprawl* compared to *Auto Innovative*, possibly owing to the densely populated areas in and a wider prevalence of transit in *Auto Innovative*, both of which increase the likelihood of mixing (as seen in the contact network structure). Notwithstanding these considerations, we find that the mean absolute percentage errors for infections and deaths across all days are largely centered around 1% (Fig. 9). This indicates that the model framework performs reasonably well and is thus potentially viable as a predictive and decision-making tool for cities in *Auto Sprawl* and *Auto Innovative*.

## Discussion

We developed a flexible and dynamic framework, PanCitySim, which is built on a modified SEIRD model combined with an activity-based mechanistic model which simulates mobility and human interactions in an urban environment. The epidemiological model enables a computation of infection dynamics of the entire population in the city during their activities (including transit) at a 5-minute resolution. We demonstrated the use of PanCitySim and the rich output it provides in two prototype cities representative of most urban areas in the US and Canada (both auto-dependent, but one with higher population density and greater share of mass transit).

We generated 5-minute activity-based contact networks for entire synthetic populations (2.5 and 4.5 million individuals in *Auto Sprawl* and *Auto Innovative*, respectively). We observed that the largest average contacts per individual is 90 in *Auto Sprawl* and 120 in *Auto Innovative*. We analyzed the degree distributions of these networks, gaining important insights into the mixing of populations across two prototype cities representative of the US and Canada. Furthermore, contact networks for four activity types were fitted. We found that these contact networks follow well-known distributions describing complex systems. Significantly, *Transit* contacts obey the power law (${\hat{\alpha }} \sim 1.4$ up to a maximum of 150 contacts), which is time-invariant and constant in two distinct city types. One implication of this is that there is no epidemic threshold for the *Transit* contact, and any number of initial infections could quickly reach epidemic proportions on this network, thus rendering it a candidate activity for early containment and intervention^62,63^. *Shopping* and *Other* follow exponential distributions that are also largely time-invariant. *Work*, on the other hand, follows a Weibull (stretched exponential) distribution with time-dependent parameters differing by city. While *Work* accounts for the greatest number of contacts per person, particularly during the middle of the day, *Transit*, more than any other activity, accounts for the closest of contacts.

Observing the dynamics of COVID-19 for 270 days in both cities, we found that even if the index cases begin on the outskirts of the city, the epidemic rapidly spreads to the city center. In both cities the epidemic peaked between days 27 and 34 with more than $9\times 10^{4}$ infections, dissipating slowly after 150 days if the city is sparse or after 250 days for a denser typology with more than $1.3\times 10^{5}$ exposed or infected individuals. Further, we found that at the onset of the epidemic it is crucial to restrict mass transit services or focus interventions (such as enforcing mask-wearing by passengers) on this activity. Post-peak, however, restrictions should be targeted towards work areas, as well as shopping centers or schools, in less dense car-oriented cities.

Our approach, which is fully mechanistic and highly spatio-temporally resolved, offers insights into the contact network structure and the importance of having a detailed representation of population mobility. With PanCitySim, scenarios can be realistically modeled and targeted to specific activities, ages, and employment types. We can detect the emergence of super-spreading events and show how urban activity patterns affects the spreading of such events. It can also be used to test distinct vaccination strategies, such as prioritizing population groups with higher exposure to the virus or higher risk of severe disease or death. The framework is responsive to changes in demand and supply availabilities and is thus useful for decision makers in understanding and mitigating epidemics in metropolitan areas.

## Supplementary information

Supplementary Information 1.

## Data Availability

The full data set used for this study is publicly available. Given that the sizes of the data files used in our experiments are large, we have created a demonstration data set with a random sample of 30,000 individuals, which can be downloaded from our Github repository for PanCitySim: https://github.com/pancitysim/PanCitySim. The repository also contains an end-to-end working Python notebook using the demonstration dataset. A supplementary Information document is also available in this repository. It comprises model implementation and calibration details, pseudocode summaries and additional implementation notes on the traffic simulator (SimMobility). An algorithmic description of the various steps and data structures involved in the implementation of PanCitySim is presented in Supplementary Section 6 (Pseudocode).
