# Supplementary material for: Activity-based epidemic propagation and contact network scaling in auto-dependent metropolitan areas
Source: Sci Rep. 2021 Nov 22;11:22665. doi: 10.1038/s41598-021-01522-w (PMC8608855; doi:10.1038/s41598-021-01522-w)
Supplement: Supplementary file 1 — Supplementary Information 1. [file 41598_2021_1522_MOESM1_ESM.pdf]

Supplementary Information: Activity-based epidemic propagation and contact network scaling in auto-dependent metropolitan areas

Authors

October 4, 2021

Contents

1 Age dependent transition probabilites for the modified SEIRD model **1**

2 Modeling contact intensity **2**
- 2.1 Distances between agents in vehicles . . . 3
- 2.2 Distances between agents performing activity at a node . . . 4
  - 2.2.1 Distances between agents staying at home . . . 5

3 Contact network generation **5**
- 3.1 Temporal split . . . 6
- 3.2 Spatial split for activities . . . 6
- 3.3 Complementary cumulative distribution functions . . . 7
- 3.4 Scaling properties . . . 7

4 Calibration **7**

5 Performance **11**

6 Pseudocode **11**

1 Age dependent transition probabilites for the modified SEIRD model

For ease of reference, we briefly represent the model and the variables representing the probability of transition between various states.

- $\boldsymbol{S}$: susceptible
- $\boldsymbol{E}$: exposed (infected, not contagious)
- $\boldsymbol{I}^S$: infectious, symptomatic (clinical)
- $\boldsymbol{I}^A$: infectious, asymptomatic (subclinical)
- $\boldsymbol{R}$: recovered
- $\boldsymbol{D}$: deceased

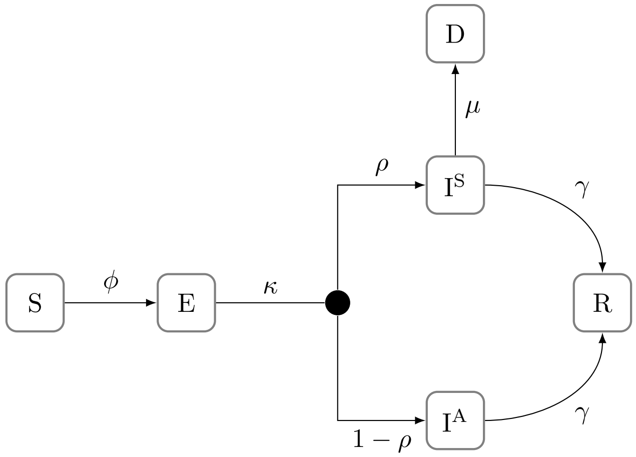

Supplementary Figure 1: Susceptible-Exposed-Infectious-Recovered-Deceased epidemiological model framework. ϕ is the transmission probability, while κ is the probability of transitioning from an exposed state to an infectious one. ρ is the age-dependent probability that an infectious individual will be symptomatic. μ is probability that an infectious individual will be deceased, while γ is the probability of recovery.

The transitions to each of these states are governed by the probabilities as shown in Supplementary Figure 1. In this section, we limit our discussion to ρ and μ since these two variables have been reported as highly age-dependent [1]. All the other transition variables (*viz.* ϕ, κ and γ) are modeled as being independent of the age of the agent Supplementary Figure 2.

We refer to Supplementary Table 1 and obtain the agent-specific value of ρ using the agent's age. Prior to starting the simulation, we use this value of ρ to mark the agents for their transition to I^A or I^S. This implies, should an agent become infected (E) during the course of the simulation, his transition to I^A or I^S is predetermined.

**Age**	$\boldsymbol{\rho}$
0-9	0.4
10-19	0.4
20-29	0.8
30-39	0.8
40-49	0.8
50-59	0.8
60-69	0.8
70-79	0.8
≥80	0.8

Supplementary Table 1: Age related values of the probability of symptomatic infectiousness, ρ, obtained from [1] based on measurements taken in China. With more granular data, these can be updated for greater detail in simulating the epidemic across various age groups.

2 Modeling contact intensity

To properly model the contact intensity, we need to estimate the mean distance between agents (persons) at each node or vehicle. For any location where interaction might occur, we model the distances between agents by assigning them random locations within the area. The vehicles and nodes are treated differently.

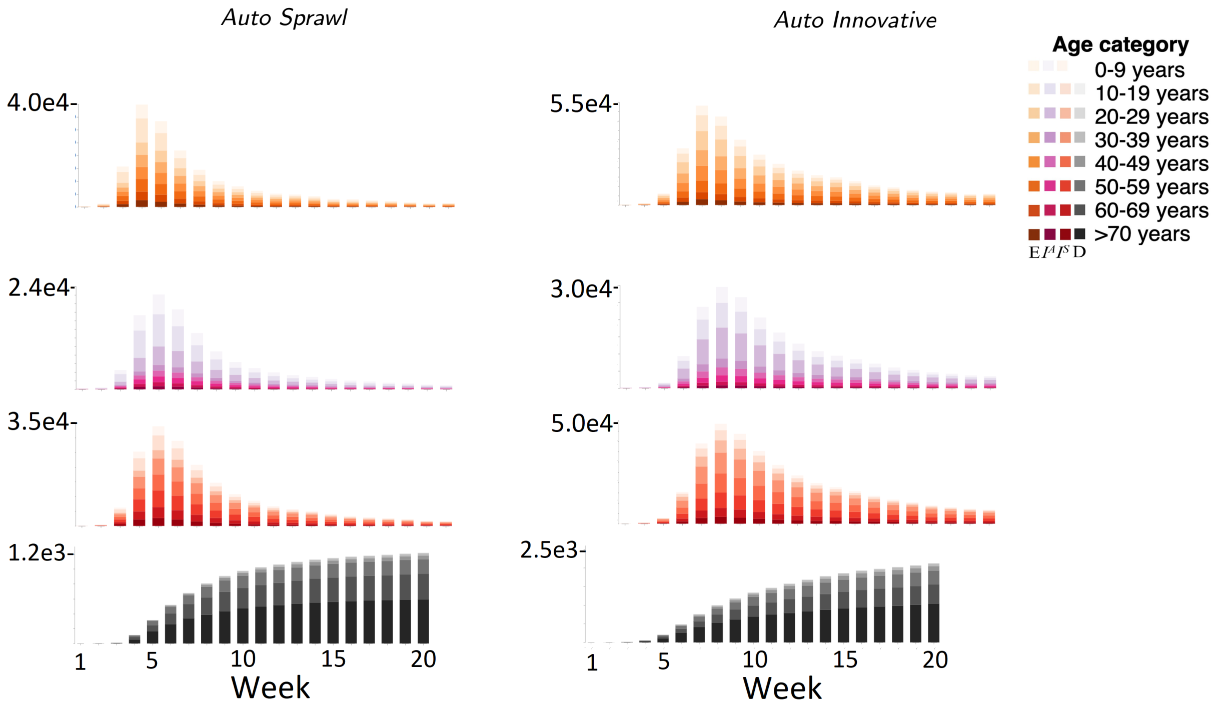

Supplementary Figure 2: Propagation of COVID-19 by age.

2.1 Distances between agents in vehicles

In the case of public transit vehicles, the shape of vehicle is always a rectangle. However, we assume the exposure to be limited to the immediate square a passenger is in. Hence, we assume the area of potential exposure to be a square of size equal to the width of the vehicle. Motivated by the data on average dimensions of vehicles, we make the following assumptions for the effective area of exposure within the vehicles:

$$A_{\text{train}} = \frac{1}{5} * \text{ [average area of train } car] \tag{1}$$

$$A_{\text{bus}} = \frac{1}{4} * \text{[average area of a bus]} \tag{2}$$

$$A_{\text{PV}} = \text{average area of a car} \tag{3}$$

Based on the assumptions made above, the effective number of infectious people to which an agent can be exposed to is also limited to the relevant sample of total number of infectious agents on the vehicle. If total number of infectious agents on a vehicle at any given time is given by $N_I[vehicle]$, the effective number($N_{EI}[vehicle]$) of infectious people can be summarised as follows:

$$N_{EI}[train] = \frac{1}{5} * \frac{1}{5} * N_I[train]; \text{assuming 5 cars in a train} \tag{4}$$

$$N_{EI}[bus] = \frac{1}{4} * N_I[bus] \tag{5}$$

$$N_{EI}[PV] = N_I[car] \tag{6}$$

$$\tag{7}$$

To each agent, we assign a random location chosen uniformly within the potential area of exposure. An illustration showing the potential exposure areas for train and buses is shown in Supplementary Figure 3. This is followed by computing the Euclidean distance between the agents based on these uniformly assigned locations. For faster implementation purposes, we use the mean of Euclidean distances instead. The use of mean distances is justified because the mean Euclidean distance between two agents converges to the

expected distance very fast with increasing number of agents as shown in Supplementary Figure 4. (See [2] for a detailed treatment of these results).

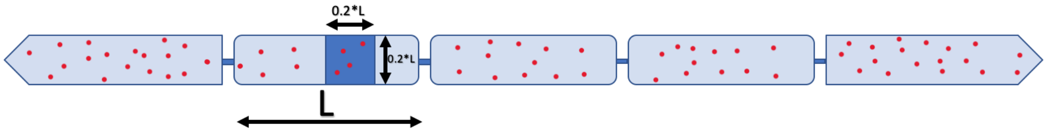

(a)

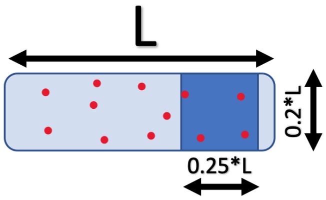

(b)

Supplementary Figure 3: Illustration showing assumptions for potential exposure areas in case of Supplementary Figure 3a: train and Supplementary Figure 3b: Bus

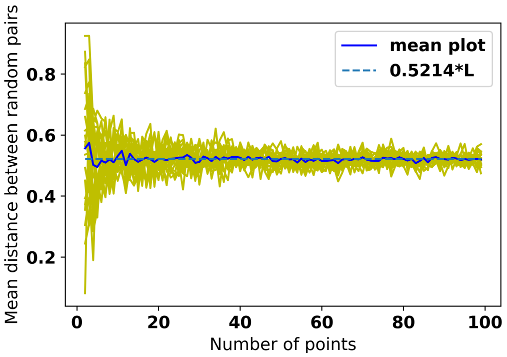

(a) Mean distance for unit square

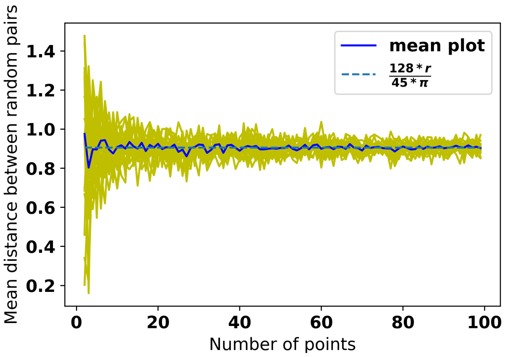

(b) Mean distance for unit circle

Supplementary Figure 4: Fast convergence of mean distance with number of points (N)

2.2 Distances between agents performing activity at a node

We partition the area of the cities into representative areas for each node of the transportation network. We use Voronoi tessellations with a clipping threshold to represent a realistic partition. The clipping threshold is selected such that post clipping, the sum of all areas, is equal to the area of the respective metros. Post clipping, a visual representation of the node-specific representative areas for both cities are shown in Supplementary Figure 5.

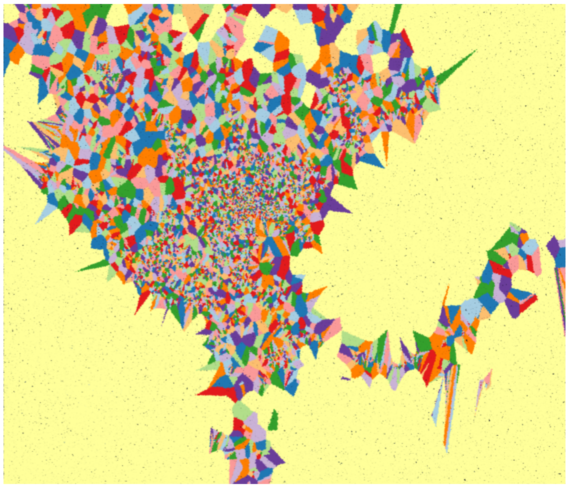

(a) *Auto Sprawl*

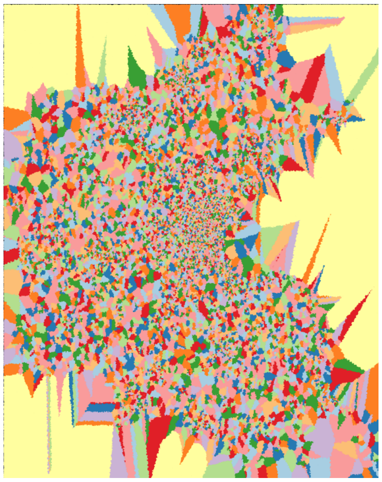

(b) *Auto Innovative*

Supplementary Figure 5: Representative areas of nodes for each city

For ease of representation, we convert these node areas to circular regions of equal as shown in Supplementary Figure 6a. Given the number of infectious agents at the respective node (N_I), we choose N_I points on the circle using a Gaussian centered at the centre of the circle. The variance of the Gaussian is proportional to the area of the circle. The proportion is chosen such that 95% of the points fall within the area of the circle (Supplementary Figure 6b). Using this relation between variance and area, we capture the idea that the agents are distributed throughout the representative area of the node. The Gaussian nature of location assignment captures the idea that hot-spot areas of the node have a higher density of agents at any given time.

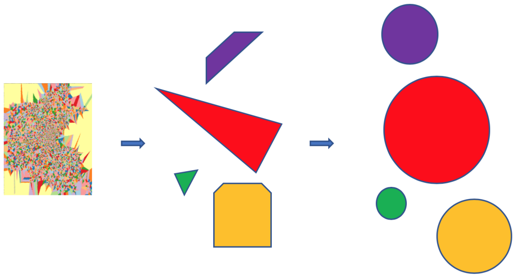

(a) Circular representations of Voronoi areas shown for three sample nodes

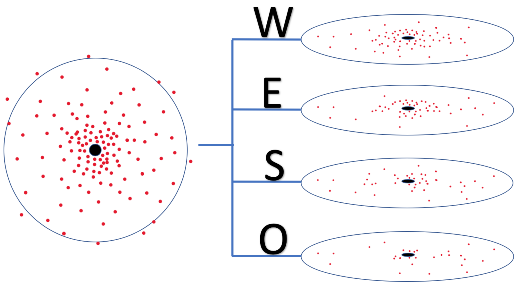

(b) Splitting of agent into layers according to activity (W: *Work*, E: *Education*, S: *Shopping*, O: *Other*)

Supplementary Figure 6: Assigning random locations to agents at a node

2.2.1 Distances between agents staying at home

The treatment of home is similar to a square vehicle of area $223\,\mathrm{m}^2$, as given by the survey from US census bureau [3].

3 Contact network generation

A contact network carries information about the frequency and intensity of contacts between the agents at different points in space and time. We accomplish the creation of the contact network from the output of

transportation simulation by separate treatment of different modes of the interaction within the network. The interaction can be either in a stationary location (e.g. a node of the city) or while traveling. Each type of interaction is a contact network. We create separate contact graphs for the three most relevant types of interactions: interactions while using public transit, interactions while performing an activity at a node of the city and interactions while staying at home. A union of these individual contact graphs gives us the full contact network for the city. We assume that the individuals who choose to drive alone, do not come into contact with anyone else during the course of the journey. Hence, we do not account for driving as an activity.

3.1 Temporal split

In order to simplify the construction of the contact network, we split the contact network into networks at different times of the day. The temporal resolution of contact network used in our simulations is 5 minutes. The contact network at any 5 minute window is a graph with several disconnected components, each component being a clique as shown in Supplementary Figure 7a. Each clique represents a location where interaction between agents might occur. At any given time all the agents present at a given location represent the nodes of the clique. The size of each clique is equal to the number of agents at the corresponding location. In order to handle large number of agents, we simplify the graph structure and transform each clique to a hub-and-spoke structure as shown in Supplementary Figure 7b. This transformation significantly reduces the number of edges in the graph and as a consequence, reduces the computing resources required to carry out the simulation. The hub-and-spoke representation is conveniently represented using the data structure discussed in Section 6.

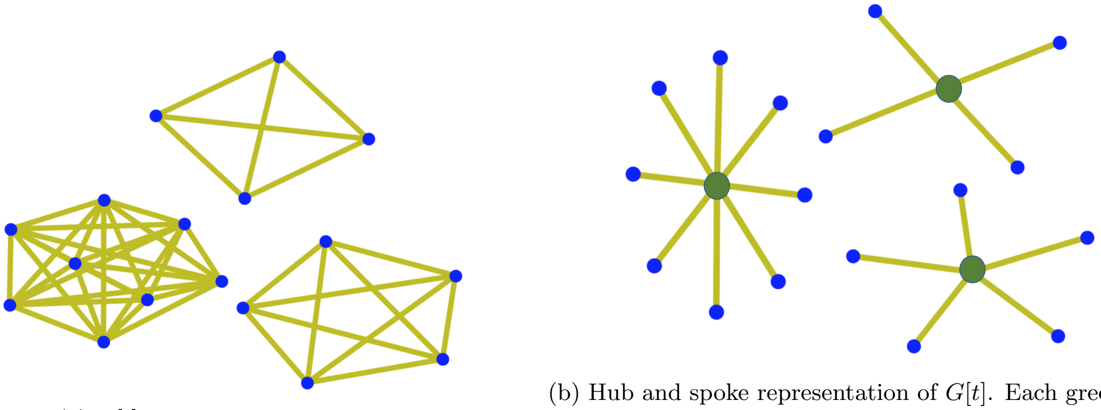

(a) $G[t]$: Contact graph at time t

(b) Hub and spoke representation of $G[t]$. Each green vertex is a dummy vertex introduced to represent a location of potential contact. (viz. a bus, train, home or a node of city)

Supplementary Figure 7: Two representations of the contact graph. The blue vertices represent agents while green vertices represent a location. The edges are not weighted in this representation and are not linked to the intensity of contact between agents.

3.2 Spatial split for activities

The construction of the contact network ensures that different locations in space are represented as a standalone component. However, the activities contact network is an exception to this. In case of the activities network, several agents present at the same node of the city may be performing a different class of activity, hence do not run a risk of interaction. In order to model this, we split the activity contact network at a node into different layers based on the type of activity as shown in Supplementary Figure 6b. Thus, only intra-layer interactions between agents are allowed.

3.3 Complementary cumulative distribution functions

We plot the complementary cumulative distribution functions (CCDFs) of the activity-specific contact networks, as well as those their union, for each city in order to explore their scaling properties Supplementary Figure 8. The CCDFs are shown for each 5-minute contact network.

3.4 Scaling properties

4 Calibration

To find Θ, we calibrate in order to achieve a basic reproductive number (average number of secondary cases caused by an infected person in the early stage of the epidemic) of $R_0 = 2.5$ using:

$$R_0 = \frac{1}{X} \sum_{m}^{X} \sum_{n}^{S} \left(1 - e^{-\Theta' \sum_m \tau_{nm}}\right) \tag{8}$$

where $\Theta' = \Theta q_0, i_0$. We use the above equation to solve for Θ'.

We notice that if we calibrate Θ' for an $R_0 = 2.5$ for a contact network generated using a sample of the population, the same Θ' does not provide a resultant $R_0 = 2.5$ when simulating with the full population. It results in very high values of R_0. We hypothesize that this behavior is due to the contact graph becoming sparse when we use a small sample of the population. As building a sample of contact network happens to be an active area of research, we leave sampling out of the scope of this work [4]. We carried out our calibration on full contact network using the entire population for both cities.

We also observed that the R_0 is not stable when using small number of initial infections(I_0). The variation in R_0 decreases as we increase I_0. This behavior is shown in Supplementary Figure 10 using three sample sizes of the population. We observe that as the population size increases, a higher value of I_0 is required to achieve a stable R_0. During calibration, we used $I_0 = 1000$ and while simulating the epidemic for 270 days, we started with an $I_0 = 200$.

(a)

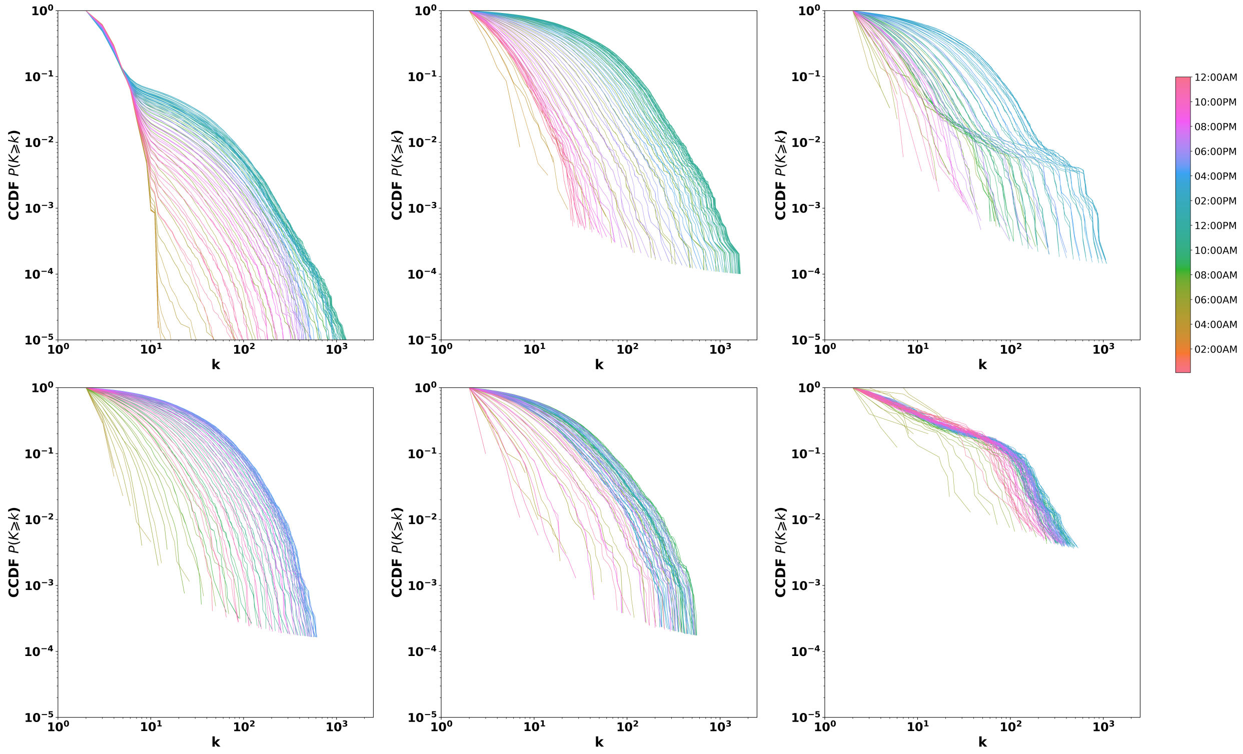

(b)

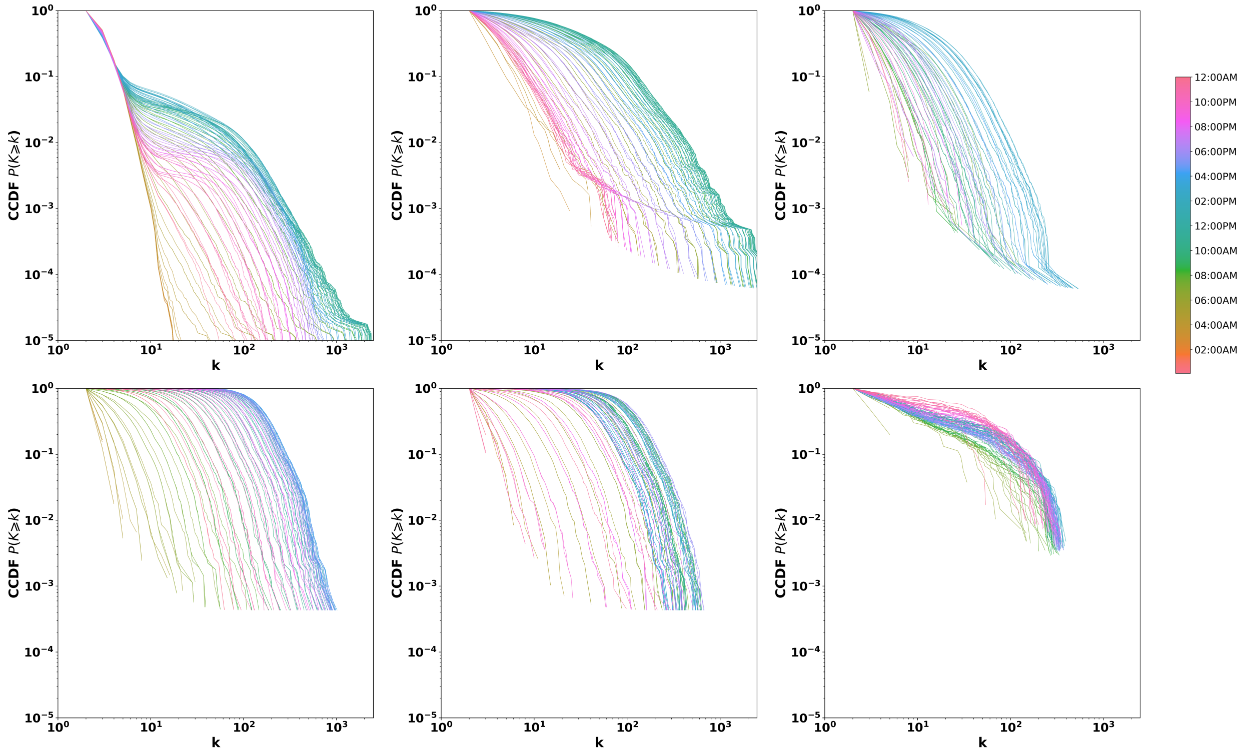

Supplementary Figure 8: **Activity-based contact networks.** Complementary cumulative distribution (CCDF) plots of time-dependent contacts (clockwise): *Union*, *Work*, *Education*, *Transit*, *Other* and *Shopping*. **a** *Auto Sprawl*; **b** *Auto Innovative.*

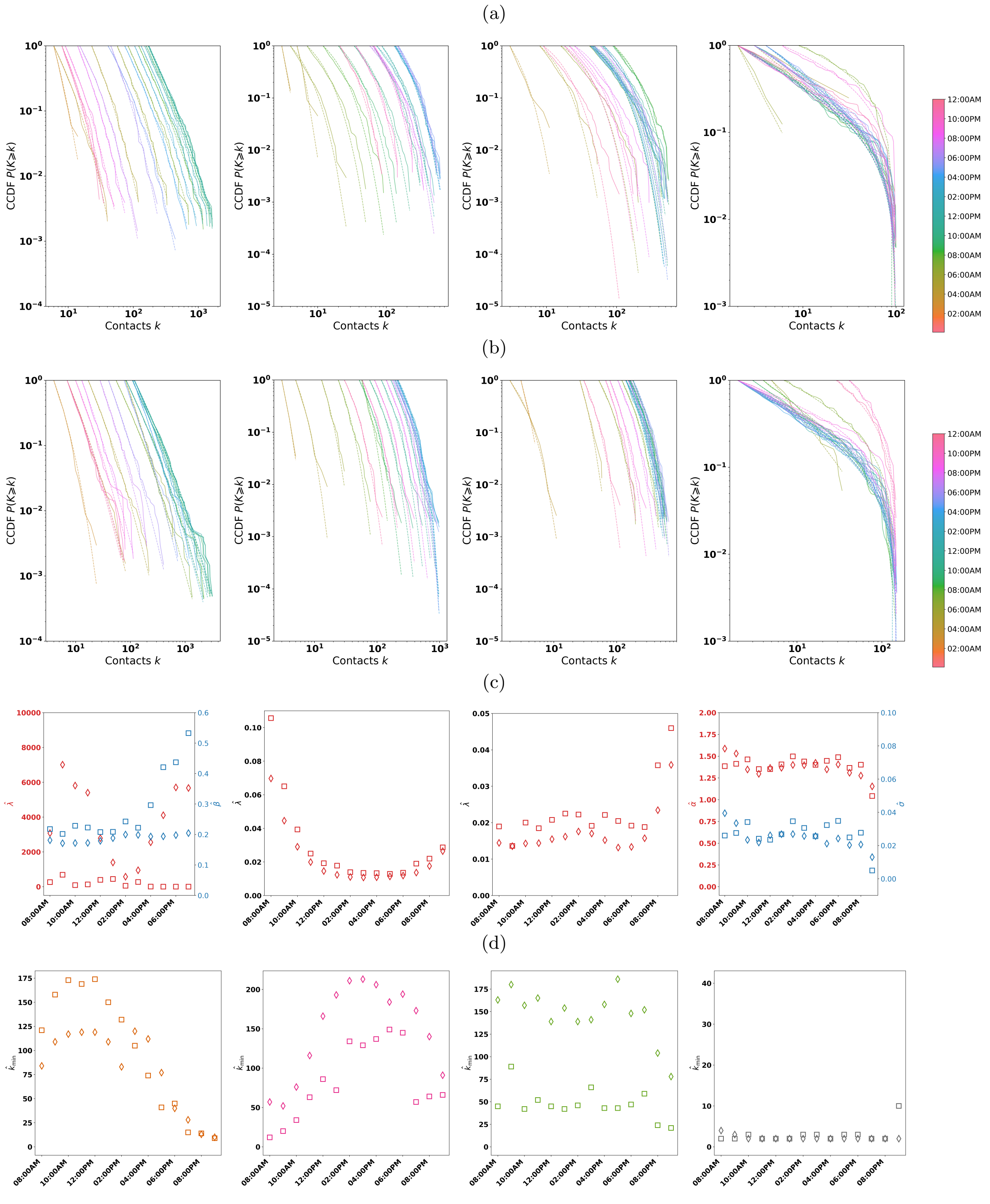

Supplementary Figure 9: **Contact network scaling.** Fitted contact network degree distributions (dashed lines) compared to observed distributions from simulation (solid lines) shown for the respective activities: *Work*, *Shopping*, *Other* and *Transit*. Only hourly fits are displayed for clarity. **a** *Auto Sprawl*; **b** *Auto Innovative*. **c** Fitted parameters for *Auto Sprawl* (square markers □) and *Auto Innovative* (diamond markers ◇). Work follows a *Weibull* distribution, while *Shopping* and *Other* are fitted to exponential distributions. *Transit* is fitted to a power law. Parameters are only significant within time periods shown. **d** Fitted $\hat{k}_{\min}$ for each activity (*Work*, *Shopping*, *Other* and *Transit*). Time-dependence is exhibited for all activities except *Transit*.

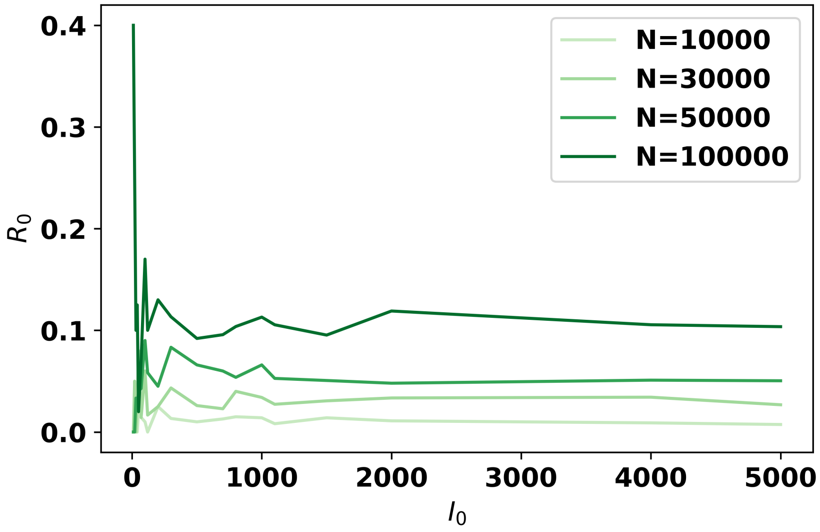

Supplementary Figure 10: Basic reproductive number R_0 versus the initial number of infected agents I_0 for a fixed values of Θ'. In order to study this behavior of R_0, we sampled three different sized sets of individuals from *Auto Sprawl* and set a small Θ'. The I_0 was chosen between 0 and 5000 with more samples for smaller values.

5 Performance

The prototyping for this study was implemented in Python. The experiments were performed an Ubuntu machine each core having a speed of around 1 GiB. In this section, we comment on the run times for the *Auto Innovative* city. The run time of the entire script can be divided into four parts. First, the pre-processing of the output files from the transport simulator (SimMobility in this case). This part takes around 5 minutes to complete. Second, the creation of three sets of contact graph; Home graph, Activity graph and Transit Graph. Each set of graphs has 288 graphs- one for each 5 minute time of the day. The creation of graphs takes around 90 minutes for a full population of 4.5 million agents in the case of *Auto Innovative.* Third, the Union operation to combine each type of individual contact graph (*Home*, *Activity* and *Transit*) takes around 10 minutes using 10 threads in parallel. Fourth, the actual simulation for a period of 270 days takes around 9 hours.

There are several avenues where the performance can be improved. First, we can parallelize the graph creation process in temporal dimension. The contact graphs at different times of the day can be generated in parallel. Second, we can parallelize the actual simulation process, with each thread processing one component of the contact graph as shown in Supplementary Figure 7b.

6 Pseudocode

Data Structure representing a contact Graph at timestep t ($G[t]$)

At every time-step t, the contact graph $G[t]$ is a set of two maps: {*forwardDicts*, *backwardDicts*}

forwardDicts is a Map $\langle$ key: $person_i$, value: $dummy_j\rangle$ `// representing an individual (`$person_i$`) who is present at a dummy location (`$dummy_j$`) at time` t

backwardDicts is a Map $\langle$ key: $dummy_j$,value: $[pid_1,\ pid_2,\ ..,\ pid_{n_j}]\rangle$ `// where` n_j `is number of individuals at the location` $dummy_j$ `at time` t

---

PanCitySim Framework

$stateVector \leftarrow (S/E/I^A/I^S/R/D)$ for every person
Initialise $stateVector[person] = S$ $\forall$ persons
$I_0 \leftarrow 200$
$\rho_{binary} \leftarrow$ use ρ from table to mark every person for a possible transition to I^S;

$day \leftarrow 1$ `// 270 days=9 months`
while $day \leq 270$ **do**
  $timestep \leftarrow 1$ `// we use 5-minute timesteps, total of 24*12=288 timesteps in a day`
  while $time - step \leq 288$ **do**
    $G \leftarrow G_{UNION[timestep]}$
    for *each dummy in G* **do**
      for *each person in* $G[backwardDict[dummy]]$ **do**
        if $stateVector[person] = I^A$ **then**
          $countInfect \leftarrow countInfect + 1$
      if $dummy = activity(W/E/S/O)$ *node* **then**
        $area \leftarrow (vornoi\ area\ of\ node(A_v))$
        $sample_{locs} \leftarrow$ ($countInfect$ locations in a circle using a Gaussian with $\sigma_x = \sigma_y = 0.0572 * A_v$)
        $reference_{loc} \leftarrow$ (1 location in a circle using a Gaussian with $\sigma_x = \sigma_y = 0.0572 * A_v$
        $mean_{dist} \leftarrow$(mean Euclidean distance between $reference_{loc}$ and $sample_{locs}$)
      if $dummy = Train$ **then**
        $mean_{dist} \leftarrow (A_{metro\ coach} * 0.2)^{0.5} * 0.5$
        $countInfect \leftarrow countInfect * \frac{1}{25}$
      if $dummy = Bus$ **then**
        $mean_{dist} \leftarrow (A_{Bus} * 0.25)^{0.5} * 0.5$
        $countInfect \leftarrow countInfect * \frac{1}{4}$
      if $dummy = Home$ **then**
        $mean_{dist} \leftarrow 6.5$
      if $countInfect > 0$ **then**
        for *each person in* $G[backwardDict[dummy]]$ **do**
          $\phi_{nt} = 1 - \exp\left(-\Theta'_{calibrated} * \frac{1}{mean^3_{dist}} * countInfect\right)$
          if $stateVector[person] = S$ **then**
            if $RAND(0,1) < \phi_{nt}$ **then**
              $stateVector[person] = E$
  for *each person in stateVector* **do**
    use γ for transitions $I^S \rightarrow R$ *and* $I^A \rightarrow R$
    use age-specific μ for transitions $I^S \rightarrow D$
    use predetermined ρ_{binary} to choose $E \rightarrow I_A$ or $E \rightarrow I_S$
    use κ to realise the transition acoording to the path chosen in previous step

References

[1] Prem K, et al. (2020) The effect of control strategies to reduce social mixing on outcomes of the COVID-19 epidemic in Wuhan, China: A modelling study. *The Lancet Public Health* 0(0).

[2] Cohen JE, Courgeau D (2017) Modeling distances between humans using Taylor's law and geometric probability. *Mathematical Population Studies* 24(4):197–218.

[3] US Census Bureau MCD (year?) Characteristics of New Housing (https://www.census.gov/construction/chars/highlights.html).

[4] Génois M, Vestergaard CL, Cattuto C, Barrat A (2015) Compensating for population sampling in simulations of epidemic spread on temporal contact networks. *Nature Communications* 6(1):8860.
